# Brief reviews

**DOI:** 10.1590/S1980-57642011DN05020014

**Published:** 2011

**Authors:** Sonia Maria Dozzi Brucki

**Affiliations:** 1 Behavioral and Cognitive Neurology Unit, Department of Neurology, and Cognitive Disorders Reference Center (CEREDIC). Hospital das Clínicas of the University of São Paulo School of Medicine, São Paulo SP, Brazil.

## SEMANTIC DEMENTIA: DEMOGRAPHY, FAMILIAL FACTORS AND SURVIVAL IN A CONSECUTIVE SERIES OF 100 CASES.

**Hodges et al. Brain 2010;133:300-306**

Authors described demographic and clinical characteristics of 100 consecutive patients seen over a 17-year period at the Memory Clinic in Cambridge.

Semantic dementia is one of the clinical variants of frontotemporal lobar degeneration, and is considered a cause of early onset dementia in most cases.

The most interesting results were: 60% men; mean age of onset of symptoms at 60.3 (7.1) years; 46% of cases had initial symptoms after the age of 65 years; 15% of cases had a positive familial history of dementia in a first degree relative; and median survival of 12.8 years. MRI predominantly showed a left predominant temporal atrophy. Twenty-four out of 32 patients had pathological confirmation, and presented ubiquitin positive and tau negative inclusions; and 13 were positive for TAR DNA binding protein (TDP-43) after re-staining.

This report yields important information about this disease in a large sample and provides clues for diagnosis: well-preserved orientation, visuospatial skills, and verbal episodic memory. With regard to impairments, these included: poor category fluency, naming and word comprehension plus surface dyslexia.

## MILD COGNITIVE IMPAIRMENT IN PARKINSON DISEASE. A MULTICENTER POOLED ANALYSIS.

**Aarsland et al. Neurology 2010;75:1062-1069.**

The aim of this study was to determine the frequency and profile of MCI in a cohort of patients with PD. Cognitive impairment is a common symptom in patients with PD, as dementia or as cognitive impairment no dementia.

The pooled data included 8 cohorts from South West Norway (2 cohorts), New York (USA), Cambridge (UK), Newcastle (UK), Philadelphia (USA), Barcelona (Spain), and Naples (Italy). Authors excluded patients with dementia or familial parkinsonism, and patients with more than 2 years of disease.

The definition for cognitive impairment was based on performance in three cognitive domains; patients were classified as having MCI if the difference between the actual cognitive domain Z-score and the expected score was below -1.5 on at least 1 out of the 3 cognitive domains.

There were 1346 patients without dementia, 25.8% being classified as MCI. Memory impairment was the most common deficit (13.3%), followed by visuospatial (11%), and attention/executive function (10.1%) impairment.

The subtypes of MCI were: 152 (11.3%) - nonamnestic MCI-single domain; 112 (8.9%) - amnestic MCI-single domain; 65 (4.8%) - amnestic MCI - multiple domains; and 18 (1.3%) - nonamnestic MCI-multiple domains.

MCI was associated with increasing age at the time of assessment and at disease onset; increasing duration of disease; severity of motor symptoms and disease stage; presence of depression, lower proportion of dopamine agonist use, and male gender.

This information can be of use in our clinical practice, particularly regarding the frequency of memory impairment.

## NEUROANATOMIC BASIS OF AMNESTIC MILD COGNITIVE IMPAIRMENT DIFFERS IN PATIENTS WITH AND WITHOUT PARKINSON DISEASE.

**Lee et al. Neurology 2010;75:2009-2016.**

Authors explored the neuroanatomic basis of amnestic mild cognitive impairment (aMCI) without Parkinson disease (PD-) and with Parkinson disease (PD+) using MRI and voxel-based morphometry (VBM).

Subjects comprised 119 consecutive patients with aMCI, classified into aMCI-PD- (n=78) and aMCI-PD+ (n=41). Groups did not differ in relation to demographic characteristics, duration of memory impairment, MMSE scores, CDR or SB (sum of boxes of the CDR). aMCI-PD- had lower scores in delayed verbal and visual recognition memory, whereas visuospatial dysfunction was more severe in patients with aMCI-PD+.

Total volume of gray matter (GM) did not differ between groups.

GM density was lower in the aMCI-PD- group in right temporal and post cingulate cortices than in controls; the aMCI-PD+ group had a lower GM density in the precuneus and left prefrontal and primary motor areas in relation to controls.

Decreased GM density in aMCI-PD- compared to aMCI-PD+ was localized in the right temporal and anterior prefrontal areas, whereas decreased GM density in AMCI-PD+ compared to aMCI-PD- involved the bilateral precuneus, primary motor and right parietal areas.

In summary, the pattern of atrophy differed between aMCI patients with and without PD. Cortical atrophy in the aMCI-PD- group was mainly localized in the temporal area, whereas atrophy in the posteromedial cortical areas (post cingulate and precuneus) was involved in aMCI-PD+ patients.

## HETEROGENEITY OF SMALL VESSEL DISEASE: A SYSTEMATIC REVIEW OF MRI AND HISTOPATHOLOGY CORRELATIONS.

**Gouw et al. J Neurol Neurosurg Psychiatry 2011;82:126-135.**

High prevalence of microbleeds, lacunes and white matter hyperintensities (WMH) are common in the elderly, and these findings are MRI expressions of cerebral small vessel disease (SVD). The aim of this paper was to characterize pathological substrates of SVD and to correlate them with postmortem MRI results. Postmortem MRI could possibly be a tool to connect the gap between MRI findings and clinical studies. Authors systematically searched for reports correlating postmortem MRI and histopathological assessment. This report gives us a profound idea of pathological findings in the SVD and its physiopathology. In the final section of the report, some techniques are suggested by authors. Quantitative MRI techniques could be better to distinguish between WMH and normal appearance white matter, such as magnetization transfer imaging and diffusion tensor imaging (DTI). Some assumptions are available from revised studies:

- **WMH:** Have presented with a sensitivity of 95% and specificity of 71% for periventricular lesions on T2 weighted postmortem MRI, which could be compared with myelin loss. For deep WMH, the sensitivity was 86% and specificity of 80%
- **Lacunes:** Few postmortem MRI studies, but numerous studies have compared lacunes with dilated perivascular spaces (Virchow-Robin). More studies are necessary to establish mechanisms of formation of both lacunes and its relationship with WMH.
- **Microbleeds:** Appear as hypo-signal on T2* MRI sequences, some reports suggest that these lesions seem to be microscopic bleedings; a minority corresponding to small lacunes, dissections of a vessel wall or to microaneurysms.
- **“Invisible” expressions of SVD:** Are areas which seem normal to postmortem MRI, but with tissue changes on pathology. For example, cortical microinfarcts (cystic or noncystic), which is an independent factor on cognitive decline in non-demented people.

More studies are necessary to establish correlations among various factors, such as microbleeds, lacunes, WMH, normal appearance WM, other degenerative pathology, and clinical pictures.

## IS PHYSICAL ACTIVITY A POTENTIAL PREVENTIVE FACTOR FOR VASCULAR DEMENTIA? A SYSTEMATIC REVIEW.

**Aarsland et al. Aging Mental Health 2011;14:386-395.**

Some evidence has been shown that physical exercise can be considered a protective factor to developing dementia. Most of studies are related to Alzheimer’s disease. In this review, authors included reports evaluating vascular dementia.

Twenty four longitudinal studies were reviewed, with 1378 patients with vascular dementia. Five studies were included in the meta-analysis, with 10.108 non-demented control subjects and 374 patients with VaD. A significant association was observed between physical exercise and a reduced risk of developing VaD: OR 0.62 (95% CI 0.42-0.92).

Physical activity should be featured as part of secondary prevention programs among people at risk of cerebrovascular disease. Reports evaluating physical exercise and prevention of cognitive decline are needed, especially those determining possible responders to exercise.

## PROGRESSIVE CHOLINERGIC DECLINE IN ALZHEIMER’S DISEASE: CONSIDERATION FOR TREATMENT WITH DONEPEZIL 23 MG IN PATIENTS WITH MODERATE TO SEVERE SYMPTOMATOLOGY.

**Sabbagh M, Cummings J. BMC Neurology 2011;11:21.**

AD has limited options in more advanced stages, which are moderate to severe dementia. In this report clinical evidence supports the use of higher than 5 to 10 mg/d doses of donepezil.

In the USA, there is a new formulation of donepezil with 23 mg, in an extended release formula. The rationale to use more elevated doses in moderate to severe stages had been based on quantity inhibition of AChE in peripheral red blood cells with conventional doses of inhibitors of cholinesterases and the most evident cholinergic deficit in patients with more severe symptoms on autopsy.

One previous double blind, randomized, head-to-head clinical trial had already showed that once-daily 23 mg of donepezil was significantly associated with better outcomes on activities of daily living and cognitive measures, as opposed to the group on a 10 mg daily regimen. Adverse events were more frequent in the highest dose group, but discontinuation was similar on both groups.

Considering that few therapeutic possibilities are available in moderate to severe group, these findings could be seen positively as an additional treatment.

## A PHASE II TRIAL OF HUPERZINE A IN MILD TO MODERATE ALZHEIMER DISEASE.

**Rafii et al. Neurology 2011;76:1389-1394.**

Huperzine A is a potent inhibitor of acetylcholinesterase and has additional effect on glutamate-induced cytotoxicity by antagonism of NMDA receptors. It is liberated for use in AD in China.

This publication shows results of a phase II study, including safety, tolerability, and efficacy of huperzine A in doses up to 200 µg BID and 400 µg BID over 16 weeks in mild to moderate AD patients.

This drug has showed to be well tolerated. But huperzine 200 µg BID did not show benefit on primary cognitive measure (ADAS-Cog), while presents positive effect on MMSE scores at 16 weeks. The highest dose had presented positive effects on ADAS-Cog and MMSE. There was no effect on ADL, NPI, and CGCI measures.

More studies are necessary to verify the efficacy of this drug and long-term treatment effects.

